# Human Papillomavirus Awareness by Educational Level and by Race and Ethnicity

**DOI:** 10.1001/jamanetworkopen.2023.43325

**Published:** 2023-11-14

**Authors:** Erica S. Stephens, Emily Dema, Jennifer K. McGee-Avila, Meredith S. Shiels, Aimée R. Kreimer, Jaimie Z. Shing

**Affiliations:** 1 Division of Cancer Epidemiology and Genetics, National Cancer Institute, Rockville, Maryland; 2 Institute for Global Health, University College London, London, United Kingdom

## Abstract

**Importance:**

Understanding disparities in human papillomavirus (HPV) awareness is crucial, given its association with vaccine uptake.

**Objective:**

To investigate differences in HPV awareness by educational attainment, race, ethnicity, and their intersectionality.

**Design, Setting, and Participants:**

This cross-sectional study used the Health Information National Trends Survey (HINTS) 5 cycles 1 to 4 data (January 26, 2017, to June 15, 2020). The data were analyzed from December 12, 2022, to June 20, 2023. A sample of the noninstitutionalized civilian US population 18 years or older was included in the analysis.

**Main Outcomes and Measures:**

Weighted prevalence of HPV awareness, HPV vaccine awareness, and knowledge that HPV causes cancer, stratified by educational attainment and by race and ethnicity. Interaction between educational attainment and race and ethnicity was assessed using a Wald test.

**Results:**

A total of 15 637 participants had educational attainment data available; of these, 51.2% were women, and the median age was 58 (IQR, 44-69) years. A total of 14 444 participants had race and ethnicity information available; of these, 4.6% were Asian, 13.9% were Black, 15.3% were Hispanic, 62.6% were White, and 3.6% were of other race or ethnicity (including American Indian or Alaska Native, Native Hawaiian or Other Pacific Islander, and more than 1 race or ethnicity). Awareness of HPV by educational attainment ranged from 40.4% for less than high school to 78.2% for college or higher; awareness by race and ethnicity ranged from 46.9% among Asian individuals to 70.2% among White individuals. Awareness of HPV vaccines across educational attainment ranged from 34.7% among those with less than high school to 74.7% among those with a college degree or higher and by race and ethnicity from 48.4% among Asian individuals to 68.2% among White individuals. Among adults who were aware of HPV, knowledge that HPV causes cervical cancer differed by educational attainment, ranging from 51.7% among those with less than high school to 84.7% among those with a college degree or higher, and by race and ethnicity, ranging from 66.0% among Black individuals to 77.9% among Asian individuals. The interaction between educational attainment and race and ethnicity on HPV awareness and HPV vaccine awareness was not significant; however, within each educational attainment level, awareness differed by race and ethnicity, with the lowest awareness consistently among Asian individuals regardless of educational attainment. Within each racial and ethnic group, HPV awareness and HPV vaccine awareness significantly decreased with decreasing educational attainment.

**Conclusions and Relevance:**

Disparities in HPV awareness were evident across social factors, with the lowest awareness among Asian individuals and individuals with lower educational attainment. These results emphasize the importance of considering social factors in HPV awareness campaigns to increase HPV vaccination.

## Key Points

**Question:**

Does human papillomavirus (HPV) awareness differ by educational attainment and race and ethnicity?

**Findings:**

In this cross-sectional study of the Health Information National Trends Survey (2017-2020) data among US adults, HPV awareness varied by educational level and by race and ethnicity. Among adults who were aware of HPV, knowledge that HPV causes cervical cancer differed by educational level and by race and ethnicity; less than one-third of the population knew HPV could cause noncervical cancers, which differed by educational level but not by race and ethnicity.

**Meaning:**

These findings suggest that educational attainment and race and ethnicity should be considered when designing HPV campaigns to expand knowledge opportunities for HPV-related cancers prevention.

## Teaser

This cross-sectional study assesses the weighted prevalence of human papillomavirus awareness by educational attainment and race and ethnicity among US noninstitutionalized adults.

## Introduction

Human papillomavirus (HPV) is the most common sexually transmitted infection in the US.^1^ Persistent high-risk HPV type infections can lead to the development of cancer at multiple anatomical sites, including the cervix, anus, vulva, vagina, penis, and oropharynx.^2^ Human papillomavirus vaccines protect against several carcinogenic HPV genotypes and constitute a well-researched, evidence-based primary prevention strategy that has the potential to reduce many HPV-driven cancers.

Despite the well-established scientific literature on HPV, the general US public lacks knowledge regarding HPV and its causality in cancers, as well as awareness of the HPV vaccine.^3,4,5^ Sociodemographic characteristics, such as identifying with a historical racial and ethnic minority group or having a lower socioeconomic status, have been associated with less HPV awareness and knowledge.^6^ This is concerning because HPV awareness and knowledge provide ample benefits, including increased involvement in primary and secondary prevention strategies such as vaccination and screening.^7,8,9,10^ Socioeconomic disparities in HPV knowledge may also be linked to systemic factors that contribute to the lack of access to health care, poorer quality of life, and higher mortality among individuals with lower educational attainment and those belonging to racial and ethnic minority communities.^3,6,11,12,13,14^

Although many US-based studies report prevalence of HPV awareness and knowledge either by educational level alone^15,16,17^ or race and ethnicity alone,^6,11,18,19,20,21,22,23^ few studies have attempted to disentangle the intersection of these highly colinear variables on HPV awareness and knowledge. Intersectionality refers to the compounding effect of several social attributes that may result in widening disparities and inequalities.^24^ It is essential to recognize that race and ethnicity, although crucial factors, serve as proxies for many unmeasured social and structural variables. These variables may encompass factors such as access to health care, socioeconomic status, cultural influences, and systemic bias, which play a pivotal role in shaping individual awareness and knowledge related to HPV.

We estimated the weighted prevalence of HPV awareness, HPV vaccine awareness, and knowledge that HPV can cause cancer, by educational attainment, race and ethnicity, and the intersection of educational attainment and race and ethnicity during 2017 to 2020. As a secondary analysis, we assessed temporal trends across survey cycles to examine whether HPV awareness and knowledge by educational attainment and race and ethnicity have improved in recent years.

## Methods

### Data Source

We used self-reported data from the Health Information National Trends Survey (HINTS), obtained by merging HINTS-5 cycles 1 to 4 (January 26, 2017, to June 15, 2020). HINTS is a nationally representative survey that is administered by the National Cancer Institute through mailed questionnaires.^25^ Participants were chosen through a complex stratified, random sampling procedure among households from a nationwide list of addresses. The survey includes adults 18 years or older in the civilian, noninstitutionalized US population. HINTS collects information on the knowledge of, attitudes toward, and use of health-related information. Regarding HPV, the survey includes questions about HPV awareness, HPV vaccine awareness, and knowledge of HPV-related cancers. We restricted the analysis to cycles 1 to 4 because these surveys included overlapping questions on HPV awareness and knowledge.

HINTS was reviewed by the Westat Institutional Review Board and was deemed exempt from review and informed consent by the US National Institutes of Health Office of Human Subjects Research Protections. Our study used publicly available data with deindividualized information and followed the Strengthening the Reporting of Observational Studies in Epidemiology (STROBE) reporting guideline. Additional information on the HINTS survey design and methodology are found elsewhere.^25^

### Primary Outcomes

Our primary outcomes were (1) awareness of HPV and HPV vaccines and (2) knowledge that HPV causes cancers of the cervix, penis, anus, and oropharynx, as measured by questions in eTable 1 in Supplement 1. Specifically, HPV awareness was assessed by the question, “Have you ever heard of HPV?” while HPV vaccine awareness was assessed by the question, “Before today, have you heard of the cervical cancer vaccine or HPV shot?” Among those who had heard of HPV (ie, were aware of HPV), knowledge of HPV-related cancers was assessed by the question, “Do you think HPV can cause (a) cervical (b) penile (c) anal (d) oral cancer?” The questionnaire did not include questions pertaining to other HPV-associated cancers (eg, vulvar and vaginal cancer). All outcomes were dichotomized (yes vs no or not sure).

### Sociodemographic Characteristics

Our main independent variables were (1) highest level of educational attainment (less than high school [<8 years of schooling or 8 through 11 years of schooling], high school graduate [12 years of schooling or completed high school], some college [including postsecondary vocational or technical schooling], and college degree or higher) and (2) self-reported race and ethnicity (Asian, Black, Hispanic, White, and other race or ethnicity [including American Indian or Alaska Native, Native Hawaiian or Other Pacific Islander, and more than 1 race or ethnicity]) (eTable 1 in Supplement 1). We combined American Indian or Alaska Native, Native Hawaiian or Other Pacific Islander, and more than 1 race or ethnicity into 1 group due to low sample sizes for these individual groups. We considered several sociodemographic characteristics self-reported by participants on the survey: age group (18-34, 35-44, 45-54, 55-64, 65-75, and ≥76 years), sex (male or female), annual household income (≤$34 999, $35 000-$74 999, and ≥$75 000), marital status (married or living as married; divorced, widowed, or separated; and single), sexual orientation (heterosexual; gay, lesbian, or bisexual; and other), and insurance status (uninsured and insured).

### Statistical Analysis

HINTS data were analyzed from December 12, 2022, to June 20, 2023. For all analyses, sampling weights were applied using the jackknife method to account for the complex sampling design of the survey.^25^ We described the distribution of sociodemographic characteristics stratified by educational attainment and by race and ethnicity. Weighted prevalence of HPV awareness and knowledge were calculated for the overall analytic population and stratified by educational attainment and by race and ethnicity. Analyses for each outcome were treated separately. Participants with missing data on educational attainment (2.8%) were excluded from the education-stratified analysis, and those with missing race and ethnicity data (10.2%) were excluded from the race and ethnicity–stratified analysis (eFigure 1 in Supplement 1). Because slightly over 10% of participants had missing race and ethnicity information, we conducted a sensitivity analysis, comparing characteristics of those with complete race and ethnicity information with all participants regardless of missing race and ethnicity data (eTable 2 in Supplement 1). The demographic characteristics were comparable between samples, indicating that our analysis did not have selection bias.

For the education-stratified analysis, weighted adjusted odds ratios (AORs) and 95% CIs were estimated using logistic regression, adjusting for confounders determined a priori, including age group, sex, marital status, and race and ethnicity. For the race and ethnicity–stratified analysis, AORs were calculated, adjusting for age group, sex, marital status, and educational attainment. To determine whether there was a linear association between educational attainment and HPV awareness and knowledge, *P* values for trend were calculated by treating educational attainment as a continuous variable. To determine whether there were significant differences in HPV awareness and knowledge across racial and ethnic groups, *P* values for heterogeneity were calculated using a Wald test.

To examine the intersection of educational attainment and race and ethnicity, we estimated weighted prevalence of HPV awareness, HPV vaccine awareness, and knowledge that HPV causes cervical cancer for each racial and ethnic group across each educational attainment level. Questions regarding penile, anal, and oral cancer were not included in the intersection analysis due to lack of power; the overall prevalence of knowledge that HPV causes noncervical cancers was low. The interaction between educational attainment and race and ethnicity on HPV awareness and knowledge outcomes was assessed using a Wald test, adjusting for age group, sex, and marital status.

Last, we estimated temporal trends in HPV awareness and knowledge for all outcomes across the survey cycle for each educational level and race and ethnicity using logistic regression, treating survey cycle (year) as a continuous variable. Due to small sample sizes of participants obtaining an educational level of less than high school after stratifying by race and ethnicity, we grouped the last educational category as high school graduate or less for both the intersection analysis and the time trends analysis. Two-sided *P* < .05 was considered statistically significant. All analyses were conducted using Stata, version 17, software (StataCorp LLC).

## Results

### Sociodemographic Characteristics

A total of 15 637 HINTS participants had data on educational attainment; of these, 51.2% (95% CI, 51.0%-51.4%) were women and 48.8% (95% CI, 48.6%-49.0%) were men (Table 1). Most of these individuals identified as heterosexual (93.6% [95% CI, 92.7%-94.4%]) and had health insurance (91.6% [95% CI, 91.4%-91.7%]). Median age was 58 (IQR, 44-69) years, which increased with decreasing levels of educational attainment. A total of 14 444 HINTS participants had data on race and ethnicity (eTable 3 in Supplement 1); of these, 4.6% (95% CI, 5.1%-5.6%) were Asian, 13.9% (95% CI, 10.6%-11.2%) were Black, 15.3% (95% CI, 16.1%-16.6%) were Hispanic, 62.6% (95% CI, 64.0%-64.7%) were White, and 3.6% (95% CI, 2.9%-3.3%) were of other race or ethnicity (including American Indian or Alaska Native, Native Hawaiian or Other Pacific Islander, and more than 1 race or ethnicity). A higher proportion of Black individuals had a household income of $34 999 or less (46.1% [95% CI, 41.9%-50.3%]) compared with other racial and ethnic groups (range, 21.3% [95% CI, 16.1%-27.5%] for Asian to 33.8% [95% CI, 30.5%-37.4%] for Hispanic). Asian participants had a lower proportion of divorced, widowed, or separated partnerships (5.6% [95% CI, 4.0%-7.9%]) compared with other racial and ethnic groups (range, 11.0% [95% CI, 8.0%-14.9%] for other race or ethnicity to 15.8% [95% CI, 13.2%-18.7%] for Black).

**Table 1.** Sociodemographic Characteristics by Educational Attainment^a^

Characteristic	Educational attainment									
	Overall (N = 15 637)		College degree or higher (n = 6987)		Some college (n = 4653)		High school graduate (n = 2898)		Less than high school (n = 1099)	
	No. of individuals^b^	Weighted % (95% CI)	No. of individuals^b^	Weighted % (95% CI)	No. of individuals^b^	Weighted % (95% CI)	No. of individuals^b^	Weighted % (95% CI)	No. of individuals^b^	Weighted % (95% CI)
Age, y (n = 15 375)										
18-34	1917	24.0 (22.8-25.2)	1132	27.8 (25.8-29.8)	489	24.6 (22.3-27.0)	230	20.2 (17.5-23.3)	66	16.8 (12.0-22.9)
35-44	1882	16.0 (15.0-17.1)	1102	20.3 (18.8-22.0)	478	14.8 (13.0-16.7)	213	12.0 (10.1-14.3)	89	16.4 (12.0-22.0)
45-54	2455	23.5 (22.5-24.6)	1219	24.2 (22.5-26.0)	688	22.9 (20.9-24.9)	391	23.1 (20.6-25.9)	157	25.2 (20.6-30.3)
55-64	3544	16.7 (16.6-16.8)	1403	13.5 (12.6-14.5)	1171	17.8 (16.8-18.8)	740	19.8 (18.3-21.5)	230	15.1 (12.5-18.1)
65-75	3661	12.5 (12.3-12.7)	1467	9.9 (9.2-10.7)	1179	13.4 (12.6-14.3)	735	14.2 (12.9-15.6)	280	13.2 (11.1-15.6)
76-104	1916	7.3 (7.1-7.5)	581	4.3 (3.8-4.8)	561	6.5 (5.9-7.2)	526	10.7 (9.6-11.8)	248	13.3 (11.1-15.9)
Median (IQR) age, y	NA	58 (44-69)	NA	54 (39-66)	NA	60 (46-69)	NA	62 (51-72)	NA	64 (51-74)
Sex (n = 15 447)										
Women	9067	51.2 (51.0-51.4)	3990	52.8 (52.6-53.1)	2650	50.5 (49.4-51.6)	1760	50.8 (48.8-52.7)	667	49.2 (44.8-53.7)
Men	6380	48.8 (48.6-49.0)	2942	47.2 (46.9-47.4)	1947	49.5 (48.4-50.6)	1088	49.2 (47.3-51.2)	403	50.8 (46.3-55.2)
Race and ethnicity (n = 14 355)										
Hispanic	2189	16.3 (16.1-16.6)	684	10.8 (9.9-11.7)	681	15.6 (14.4-16.8)	459	19.0 (17.0-21.1)	365	35.8 (31.0-41.0)
Asian	657	5.3 (5.1-5.6)	455	9.6 (8.8-10.6)	118	3.3 (2.5-4.4)	52	2.9 (2.0-4.1)	32	4.2 (2.5-6.9)
Black	1996	10.9 (10.6-11.2)	743	8.8 (8.0-9.7)	662	9.7 (8.8-10.8)	426	14.0 (12.5-15.7)	165	16.5 (12.6-21.2)
Hispanic	2189	16.3 (16.1-16.6)	684	10.8 (9.9-11.7)	681	15.6 (14.4-16.8)	459	19.0 (17.0-21.1)	365	35.8 (31.0-41.0)
White	8994	64.4 (64.0-64.7)	4556	68.2 (67.9-68.6)	2642	68.2 (67.0-69.4)	1483	60.1 (57.8-62.3)	313	41.1 (36.2-46.1)
Other^c^	519	3.1 (2.9-3.3)	228	2.5 (2.0-3.2)	171	3.2 (2.5-4.0)	82	4.1 (2.9-5.8)	38	2.5 (1.5-4.0)
Annual household income (n = 15 458)										
≥$75 000	5775	39.2 (37.9-40.6)	3899	61.8 (60.0-63.6)	1354	37.3 (34.9-39.8)	455	21.2 (18.7-24.0)	67	12.2 (8.0-18.1)
$35 000-$74 999	4714	31.5 (30.1-33.0)	2001	25.5 (24.0-27.2)	1570	35.1 (32.8-37.6)	934	36.0 (33.2-38.8)	209	25.3 (20.5-30.8)
$0-$34 999	4969	29.2 (28.0-30.5)	1019	12.6 (11.2-14.2)	1678	27.6 (25.6-29.6)	1477	42.8 (39.9-45.8)	795	62.5 (56.2-68.4)
Marital status (n = 15 527)										
Divorced, widowed, or separated	4609	15.0 (14.6-15.5)	1491	8.9 (8.2-9.6)	1533	15.5 (14.5-16.6)	1117	19.8 (18.2-21.4)	468	23.0 (19.0-27.5)
Married or living as married	8292	54.5 (54.0-55.0)	4227	61.8 (59.9-63.6)	2317	52.5 (50.6-54.4)	1309	50.7 (47.9-53.5)	439	47.2 (42.3-52.0)
Single	2626	30.5 (30.2-30.7)	1231	29.3 (27.4-31.4)	774	32.0 (30.0-34.1)	440	29.6 (26.7-32.5)	181	29.9 (24.7-35.6)
Sexual orientation (n = 14 983)										
Gay, lesbian, or bisexual	597	4.8 (4.1-5.6)	336	5.6 (4.8-6.7)	171	4.8 (3.8-6.2)	65	3.4 (2.3-5.0)	25	5.0 (2.4-10.0)
Heterosexual	14 171	93.6 (92.7-94.4)	6433	93.4 (92.3-94.3)	4263	93.6 (92.1-94.9)	2563	94.7 (92.4-96.3)	912	91.7 (87.2-94.7)
Other	215	1.6 (1.2-2.1)	72	1.0 (0.6-1.5)	56	1.5 (1.0-2.4)	47	1.9 (1.1-3.5)	40	3.3 (2.2-5.0)
Insurance status (n = 15 420)										
Uninsured	789	8.4 (8.3-8.6)	194	4.0 (3.1-5.2)	277	8.5 (7.2-10.0)	204	10.2 (8.5-12.3)	114	19.9 (15.1-25.8)
Insured	14 631	91.6 (91.4-91.7)	6715	96.0 (94.8-96.9)	4313	91.5 (90.0-92.8)	2646	89.8 (87.7-91.5)	957	80.1 (74.2-84.9)

Abbreviation: NA, not applicable.

^a^ Data are from Health Information Trends Survey 5 cycles 1 to 4 (2017-2020).

^b^ Indicates the total number of individuals in each population of interest (eg, all individuals with college degree or higher, all individuals with some college).

^c^ Includes American Indian or Alaska Native, Native Hawaiian or Other Pacific Islander, and more than 1 race or ethnicity.

### HPV Awareness and Knowledge by Educational Attainment

**Figure 1.**
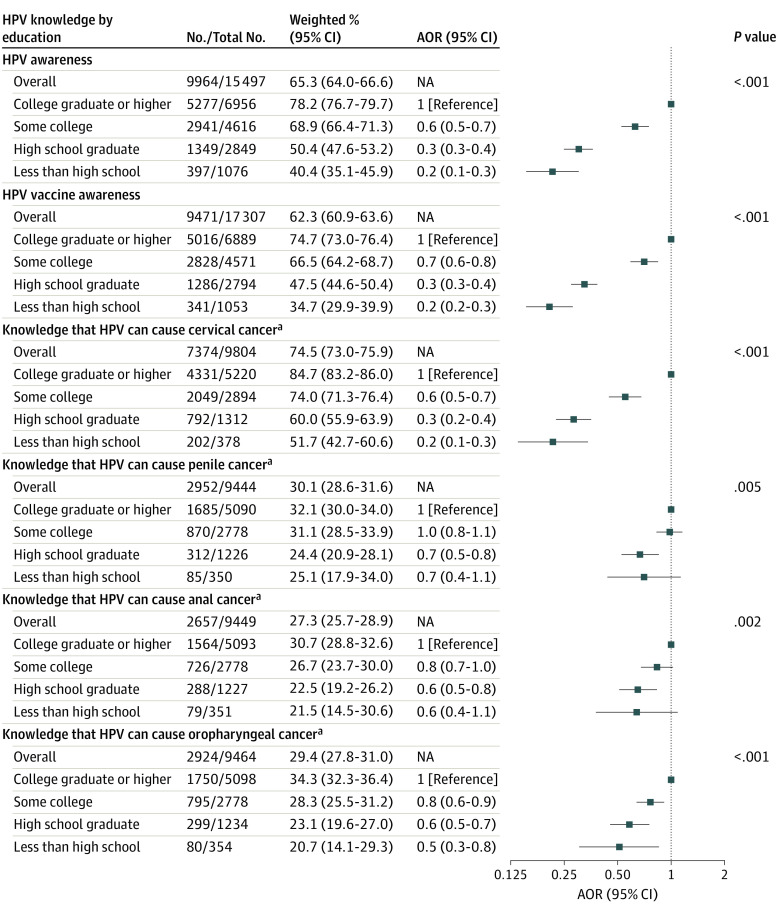
Weighted Prevalence and Adjusted Odds Ratios (AORs) of Human Papillomavirus (HPV) Awareness and Knowledge by Educational Attainment, 2017 to 2020 No. indicates the number of individuals with the outcome of interest (eg, responded “yes” to having heard of HPV); total No., individuals in each population of interest for each outcome (eg, all individuals who obtained a college degree or higher and responded to the HPV awareness question), excluding any missing responses for each question (missing data ranged from 1% to 5% depending on the survey question). The ORs were adjusted for age group, sex, marital status, and race and ethnicity. NA indicates not applicable. ^a^Includes only individuals who were aware of HPV.

**Figure 2.**
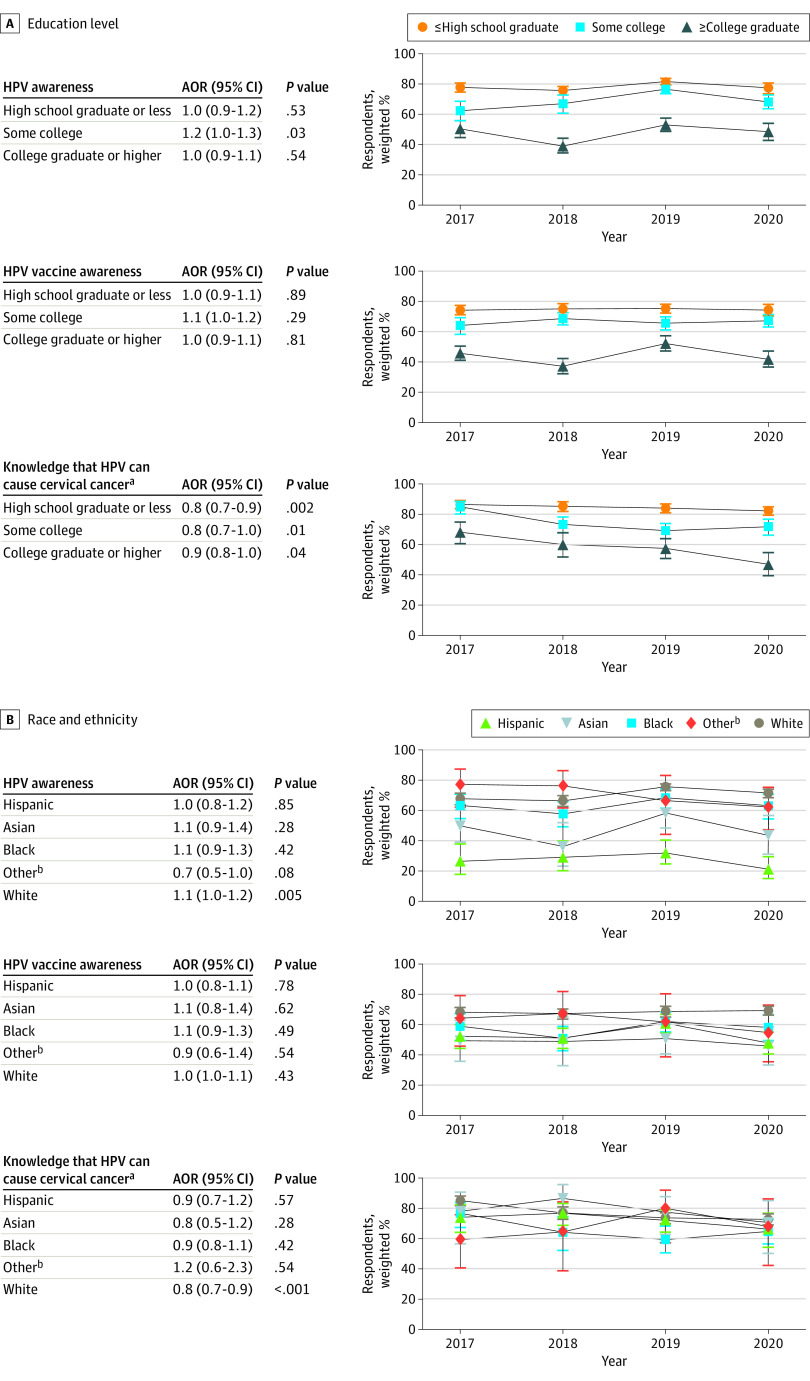
Trends in Human Papillomavirus (HPV) Awareness and Knowledge by Educational Attainment and Race and Ethnicity, 2017 to 2020 Adjusted odds ratios (AORs) indicate the relative change in prevalence per year. ^a^Includes only individuals who were aware of HPV. ^b^Includes American Indian or Alaska Native, Native Hawaiian or Other Pacific Islander, and more than 1 race or ethnicity.

Awareness of HPV decreased with decreasing educational attainment (*P* < .001 for trend), from 78.2% (95% CI, 76.7%-79.7%) for individuals with a college degree or higher to 40.4% (95% CI, 35.1%-45.9%) for individuals with less than high school (Figure 1). Compared with individuals with a college degree or higher, individuals with some college education had 40% lower odds (AOR, 0.6 [95% CI, 0.5-0.7]), high school graduates had 70% lower odds (AOR, 0.3 [95% CI, 0.3-0.4]), and individuals with less than a high school education had 80% lower odds (AOR, 0.2 [95% CI, 0.1-0.3]) of HPV awareness. A similar pattern was observed for HPV vaccine awareness, which ranged by educational level from 34.7% (95% CI,29.9%-39.9%) among those with less than high school to 74.7% (95% CI, 73.0%-76.4%) among those with a college degree or higher. Temporal trends in HPV awareness across survey cycle only significantly increased among individuals with some college education (AOR, 1.2 [95% CI, 1.0-1.3]; *P* = .03 for trend); HPV vaccine awareness remained stable across survey cycles for all educational attainment levels. (Figure 2). Among adults who were aware of HPV, 74.5% (95% CI, 73.0%-75.9%) knew that HPV causes cervical cancer, which differed by educational attainment, ranging from 51.7% (95% CI, 42.7%-60.6%) among those who did not complete high school to 84.7% (95% CI, 83.2%-86.0%) among those with a college degree or higher. Knowledge that HPV causes other cancer types was low: 30.1% (95% CI, 28.6%-31.6%) for penile cancer, 27.3% (95% CI, 25.7%-28.9%) for anal cancer, and 29.4% (95% CI, 27.8%-31.0%) for oropharyngeal cancer (Figure 1). Knowledge decreased with decreasing educational attainment for all cancer types (eg, 21.5% [95% CI, 14.5%-30.6%] of individuals with less than high school compared with 30.7% [95% CI, 28.8%-32.6%] of those with a college degree or higher knew HPV can cause anal cancer) (*P* = .002 for trend). Across survey cycles, knowledge that HPV causes cervical cancer significantly declined over time for all educational levels, most notably for individuals with a high school education or less (AOR, 0.8 [95% CI, 0.7-0.9]; *P* = .002 for trend). (Figure 2). Knowledge that HPV causes penile, anal, and oropharyngeal cancer remained stable and low over time for all educational attainment levels (eFigure 2 in Supplement 1).

### HPV Awareness and Knowledge by Race and Ethnicity

**Figure 3.**
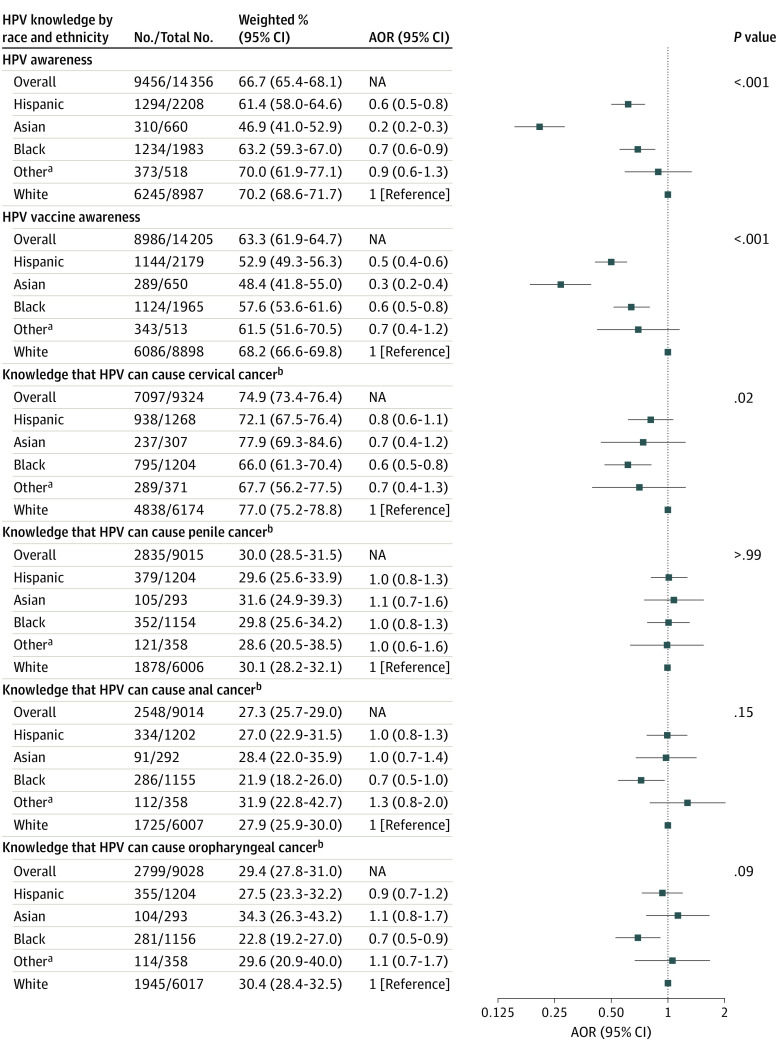
Race and Ethnicity–Stratified Weighted Prevalence and Adjusted Odds Ratios (AORs) of Human Papillomavirus (HPV) Awareness and Knowledge, 2017 to 2020 No. indicates the number of individuals with the outcome of interest (eg, responded “yes” to having heard of HPV); total No., individuals in each population of interest for each outcome (eg, all individuals who obtained a college degree or higher and responded to the HPV awareness question), excluding any missing responses for each question (missing data ranged from 1% to 5% depending on the survey question). The ORs were adjusted for age group, sex, marital status, and education. NA indicates not applicable. ^a^Includes American Indian or Alaska Native, Native Hawaiian or Other Pacific Islander, and more than 1 race or ethnicity. ^b^Includes only individuals who were aware of HPV.

Awareness of HPV differed by race and ethnicity (*P* < .001 for heterogeneity), ranging from 46.9% (95% CI, 41.0%-52.9%) for Asian individuals to 70.2% (95% CI, 68.6%-71.7%) for White individuals (Figure 3). Compared with White individuals, Hispanic individuals had 40% lower odds (AOR, 0.6 [95% CI, 0.5-0.8]), Asian adults had 80% lower odds (AOR, 0.2 [95% CI, 0.2-0.3]), and Black adults had 30% lower odds (AOR, 0.7 [95% CI, 0.6-0.9]) of HPV awareness. A similar pattern was observed for HPV vaccine awareness, which ranged by race and ethnicity from 48.4% (95% CI, 41.8%-55.0%) among Asian individuals to 68.2% (95% CI, 66.6%-69.8%) among White individuals. Temporal trends in HPV awareness across survey cycles remained stable for all racial and ethnic groups except White adults, for whom HPV awareness significantly increased (AOR, 1.1 [95% CI, 1.0-1.2]; *P* = .005 for trend) (Figure 2). Among adults who were aware of HPV, knowledge that HPV causes cervical cancer differed across racial and ethnic groups (*P* = .02 for heterogeneity), ranging from 66.0% (95% CI, 61.6%-70.4%) for Black adults to 77.9% (95% CI, 69.3%-84.6%) for Asian adults (Figure 3). Among adults who were aware of HPV, knowledge that HPV causes penile, anal, and oropharyngeal cancer was low and did not differ by racial and ethnic groups (eg, range for anal cancer, 21.9% [95% CI, 18.2%-26.0%] for Black adults to 31.9% [95% CI, 22.8%-42.7%] for adults of other race or ethnicity; *P* = .15 for heterogeneity) (Figure 3) and remained stable over time (eFigure 2 in Supplement 1).

### Intersectionality of Educational Attainment and Race and Ethnicity

The interactions between educational attainment and race and ethnicity on HPV awareness, HPV vaccine awareness, and knowledge that HPV causes cervical cancer were not significant; however, disparities in HPV awareness and knowledge still existed across each intersection (Table 2). Specifically, among each educational level, HPV awareness and HPV vaccine awareness differed by race and ethnicity, with the lowest awareness consistently among Asian individuals regardless of educational attainment (eg, 27.4% [95% CI, 14.0%-46.7%] for HPV awareness for those attaining a high school education or less); for example, among individuals with some college education, 38.0% (95% CI, 23.4%-55.1%) of Asian individuals were aware of HPV compared with 71.5% (95% CI, 68.5%-74.4%) of White individuals (*P* < .001 for heterogeneity). Among each racial and ethnic group, lower levels of educational attainment were associated with less HPV awareness and HPV vaccine awareness; for example, among Hispanic individuals, 80.5% (95% CI, 75.8%-84.4%) with a college degree or higher were aware of HPV compared with 45.0% (95% CI, 39.7%-50.5%) with a high school education or less (*P* < .001 for trend). Among adults who were aware of HPV, knowledge that HPV can cause cervical cancer differed by race and ethnicity among those with a college degree or higher (range, 77.6% [95% CI, 60.1%-88.9%] for other race or ethnicity to 86.1% [95% CI, 84.4%-87.6%] for White; *P* = .007 for heterogeneity) and those with some college education (range, 62.5% [95% CI, 54.3%-70.0%] for Black to 76.3% [95% CI, 73.1%-79.3%] for White; *P* = .004 for heterogeneity) but did not differ by race and ethnicity among participants with a high school education or less (range, 55.3% [95% CI, 32.3%-76.3%] for other race or ethnicity to 66.6% [95% CI, 22.1%-93.3%] for Asian; *P* = .98 for heterogeneity). Among each racial and ethnic group, lower levels of educational attainment were associated with less knowledge that HPV can cause cervical cancer (*P* < .05 for trend), except for Asian participants and participants of other race or ethnicity.

**Table 2.** Weighted Prevalence of HPV Knowledge by Race and Ethnicity Across Educational Attainment^a^

Race and ethnicity	Educational attainment						*P* value for trend
	College degree or higher		Some college		High school graduate or less		
	No./total No.^b^	Weighted % (95% CI)	No./total No.^b^	Weighted % (95% CI)	No./total No.^b^	Weighted % (95% CI)	
**HPV awareness (*P* value for interaction = .37)**							
Asian	245/454	56.8 (50.0-63.4)	51/118	38.0 (23.4-55.1)	13/84	27.4 (14.0-46.7)	.008
Black	540/739	73.4 (66.3-79.5)	419/652	69.7 (64.3-74.6)	265/579	50.5 (43.5-57.5)	<.001
Hispanic	517/683	80.5 (75.8-84.4)	432/679	69.2 (63.5-74.5)	333/821	45.0 (39.7-50.5)	<.001
White	3587/4540	82.0 (80.2-83.6)	1750/2627	71.5 (68.5-74.4)	886/1778	52.2 (48.3-56.0)	<.001
Other^c^	195/227	86.8 (74.6-93.7)	115/171	73.9 (60.6-84.0)	63/119	53.6 (38.3-68.2)	.003
*P* value for heterogeneity	NA	<.001	NA	<.001	NA	<.001	NA
**HPV vaccine awareness (*P* value for interaction = .24)**							
Asian	228/449	55.5 (48.5-62.3)	45/117	46.5 (28.8-65.0)	16/81	28.4 (15.4-46.5)	.005
Black	511/735	69.8 (62.5-76.3)	366/646	58.4 (52.3-64.2)	237/571	48.3 (41.3-55.4)	.003
Hispanic	461/677	69.8 (63.8-75.2)	389/677	60.0 (53.9-65.8)	284/801	38.1 (32.6-43.8)	<.001
White	3459/4498	79.5 (77.7-81.2)	1743/2603	71.2 (68.2-74.0)	858/1756	48.1 (44.6-51.6)	<.001
Other^c^	178/225	77.4 (62.7-87.5)	106/170	60.7 (44.7-74.7)	59/117	51.4 (34.4-68.0)	.13
*P* value for heterogeneity	NA	<.001	NA	<.001	NA	<.001	NA
**Knowledge that HPV can cause cervical cancer**^d^ **(*P* value for interaction = .73)**							
Asian	192/242	83.6 (76.0-89.2)	36/51	63.0 (36.7-83.3)	DS	66.6 (22.1-93.3)	.22
Black	407/529	79.6 (73.0-84.9)	242/410	62.5 (54.3-70.0)	138/256	56.6 (45.7-66.9)	.002
Hispanic	424/511	82.4 (76.0-87.4)	319/428	75.3 (67.9-81.5)	189/317	58.3 (48.9-67.2)	<.001
White	3007 /3557	86.1 (84.4-87.6)	1274/1724	76.3 (73.1-79.3)	DS	59.3 (54.1-64.3)	<.001
Other^c^	34/228	77.6 (60.1-88.9)	85/114	67.9 (46.7-83.6)	41/63	55.3 (32.3-76.3)	.28
*P* value for heterogeneity	NA	.007	NA	.004	NA	.98	NA

Abbreviations: DS, data suppressed (n <10); HPV, human papillomavirus; NA, not applicable.

^a^ Data are from Health Information Trends Survey 5 cycles 1 to 4 (2017-2020).

^b^ Indicates number of individuals with the outcome of interest (eg, responded “yes” to having heard of HPV)/total number of individuals in each population of interest for each outcome (eg, all Hispanic individuals who obtained a college degree or higher and responded to the HPV awareness question).

^c^ Includes American Indian or Alaska Native, Native Hawaiian or Other Pacific Islander, and more than 1 race or ethnicity.

^d^ Among individuals who were aware of HPV.

## Discussion

In this large, nationally representative cross-sectional study of US adults, we observed significant differences in HPV awareness and knowledge by levels of educational attainment and race and ethnicity during 2017 to 2020. Individuals with lower educational attainment and those from a minority racial and ethnic group were less aware of HPV and the HPV vaccine compared with those with higher educational attainment and White individuals. While a large proportion of adults who were aware of HPV knew HPV could cause cervical cancer (74.5%), less than one-third of individuals knew HPV could cause noncervical cancers. Additionally, knowledge of HPV’s causal role in noncervical cancers has not improved over time during recent survey cycles for any level of educational attainment or race and ethnicity.

Our results corroborate studies that have demonstrated approximately 65% of US adults are aware of HPV and the HPV vaccine, and among those who are aware of HPV, approximately 70% know that HPV can cause cervical cancer.^14,22,26,27^ Studies have also demonstrated very low prevalence of knowledge that HPV causes penile, anal, and oropharyngeal cancer (approximately 30%).^14,22,26,27^ To expand on the foundation set by these prior studies among adults overall, our study more granularly investigates knowledge differences across sociodemographic groups to elucidate knowledge gaps and disparities. Specifically, HPV vaccine awareness was as low as 34.7% among individuals with less than high school education compared with 74.7% among individuals with a college degree or higher; these differences were masked by overall estimates of HPV vaccine awareness. By race and ethnicity, HPV vaccine awareness was as low as 48.4% among Asian individuals compared with 68.2% among White individuals. Most notably, when looking within cross-strata of educational attainment and race and ethnicity, only 27.4% of Asian individuals attaining a high school education were aware of the HPV vaccine, which emphasizes the importance of considering multiple identities to adequately address population-level knowledge gaps and heath disparities.

Our results highlight the compounding effects of educational attainment and race and ethnicity. However, social constructs such as race and ethnicity also serve as proxies for outcomes such as lower educational attainment due to the influence of historical and pervasive oppression, including institutional and systemic racism. These forms of discrimination are deeply embedded into US society and have perpetrated unequal allocation of resources such as quality health care, education, and economic opportunities for racial and ethnic minority groups.^28,29^ Our results suggest that this systemic issue may have contributed to observed disparities in HPV awareness and knowledge among individuals with lower educational attainment and individuals from racial and ethnic minority backgrounds. Educational attainment and racial and ethnic identification are highly correlated through mechanisms rooted in structural racism.^30,31^ Historical and commemoratory injustices in education, including disparities in school funding, biased disciplinary practices, and unequal resource allocation, have created substantial barriers to educational attainment for some racial and ethnic groups.^30,31^ Studies show the importance of access to community health and resource centers that provide access to education, financial resources, and pathways for HPV vaccination and risk prevention.^10,32,33^ By acknowledging the compounding impact of educational disparities and structural racism, we can better understand how to disseminate resources equitably among groups with less HPV awareness and knowledge.

We found no improvement in knowledge of HPV’s causal role in cancer, which presents a pressing need to improve education surrounding HPV-associated cancers. Specifically, knowledge that HPV can cause noncervical cancers did not significantly change from 2017 to 2020 for all educational attainment levels and racial and ethnic groups. Although it has been well-established that 90% of anal cancers are attributed to HPV,^34^ only 1 in 4 US adults attaining a high school education or less who were aware of HPV knew that HPV could cause anal cancer. Currently, national screening recommendations are not available for noncervical HPV-driven cancers, such as anal and oropharyngeal cancer, which may contribute to the lack of knowledge and awareness about these cancers in general.

### Strengths and Limitations

A major strength of our study was the survey’s large, nationally representative sample. Several cycles were merged to increase power and provide more stable estimates, especially given that many studies of HPV awareness and knowledge by educational attainment or race and ethnicity only examined a single survey cycle and were limited in sample size. Additionally, substantial efforts were made to reduce potential selection bias in the HINTS methodology through complex sampling and weighting. To our knowledge, our study is the first to disentangle the association of highly correlated variables (educational attainment and race and ethnicity) with HPV awareness and knowledge and to describe more recent trends in HPV awareness and knowledge over time by educational attainment and race and ethnicity.

This study has some limitations. Survey response rates for HINTS-5 were low, with rates of 32.4% (cycle 1, 2017), 32.9% (cycle 2, 2018), 30.3% (cycle 3, 2019), and 36.7% (cycle 4, 2020), and these low response rates may have resulted in the poorer capture of racial and ethnic minority groups, such as individuals identifying as Hispanic or African American, as noted in the HINTS methodology.^25^ Additionally, we were unable to separately examine some historically underrepresented races (eg, American Indian or Alaska Native individuals and Native Hawaiian and Other Pacific Islander individuals) and more granular groups of ethnicity (eg, Asian Indian, Chinese, Filipino) due to low sample sizes. Furthermore, the language in the questionnaires was not entirely consistent across surveys. For example, to capture gender, the HINTS-5 cycles 1 to 3 asked, “Are you male or female?” while HINTS-5 cycle 4 asked, “On your original birth certificate, were you listed as male or female?” This language shift may have resulted in the overlap of sex and gender, resulting in inaccurate representation of distributions by identity.

## Conclusions

In this cross-sectional study, we highlight important differences in HPV awareness and knowledge among US adults with lower educational attainment and among racial and ethnic minority groups, suggesting 2 potential issues: (1) inequitable dissemination of information and (2) a need to tailor messages regarding HPV-related information. Educational attainment and race and ethnicity should be considered when disseminating HPV awareness campaigns to expand equitable opportunities to reduce HPV knowledge disparities. Because vaccination is a highly researched strategy to prevent HPV-driven cancers, increasing HPV awareness and knowledge allows individuals to better understand their health and factors regarding health-related decision-making. If HPV awareness and knowledge can be expanded and improved among adults, they may be more likely to obtain vaccination for their children during age-appropriate periods.

## Notes

Supplement 1. **eTable 1.** Survey Questions Used to Ascertain Study Measures, Health Information Trends Survey 5 Cycles 1 to 4 (2017-2020) **eTable 2.** Comparison of Participants With and Without Data on Race and Ethnicity **eTable 3.** Sociodemographic Characteristics by Race and Ethnicity, Health Information Trends Survey 5 Cycles 1 to 4 (2017-2020) **eFigure 1.** Data Inclusion by Educational Level and Race and Ethnicity, Health Information Trends Survey 5 Cycles 1 to 4 (2017-2020) **eFigure 2.** Trends in Knowledge that HPV Can Cause Penile, Anal, and Oropharyngeal Cancer Among Participants Who Were Aware of HPV, Health Information Trends Survey 5 Cycles 1 to 4 (2017-2020)

Supplement 2.
Data Sharing Statement
