# Supplementary material for: Human Papillomavirus Awareness by Educational Level and by Race and Ethnicity
Source: JAMA Netw Open. 2023 Nov 14;6(11):e2343325. doi: 10.1001/jamanetworkopen.2023.43325 (PMC10646733; doi:10.1001/jamanetworkopen.2023.43325)
Supplement: Supplement 1. — eTable 1. Survey Questions Used to Ascertain Study Measures, Health Information Trends Survey 5 Cycles 1 to 4 (2017-2020) eTable 2. Comparison of Participants With and Without Data on Race and Ethnicity eTable 3. Sociodemographic Characteristics by Race and Ethnicity, Health Information Trends Survey 5 Cycles 1 to 4 (2017-2020) eFigure 1. Data Inclusion by Educational Level and Race and Ethnicity, Health Information Trends Survey 5 Cycles 1 to 4 (2017-2020) eFigure 2. Trends in Knowledge that HPV Can Cause Penile, Anal, and Oropharyngeal Cancer Among Participants Who Were Aware of HPV, Health Information Trends Survey 5 Cycles 1 to 4 (2017-2020) [file jamanetwopen-e2343325-s001.pdf]

Supplementary Online Content

Stephens ES, Dema E, McGee-Avila JK, Shiels MS, Kreimer AR, Shing JZ. Human papillomavirus awareness by educational level and by race and ethnicity. *JAMA Netw Open*. 2023;6(11):e2343325. doi:10.1001/jamanetworkopen.2023.43325

eTable 1. Survey Questions Used to Ascertain Study Measures, Health Information Trends Survey 5 Cycles 1 to 4 (2017-2020)
eTable 2. Comparison of Participants With and Without Data on Race and Ethnicity
eTable 3. Sociodemographic Characteristics by Race and Ethnicity, Health Information Trends Survey 5 Cycles 1 to 4 (2017-2020)
eFigure 1. Data Inclusion by Educational Level and Race and Ethnicity, Health Information Trends Survey 5 Cycles 1 to 4 (2017-2020)
eFigure 2. Trends in Knowledge that HPV Can Cause Penile, Anal, and Oropharyngeal Cancer Among Participants Who Were Aware of HPV, Health Information Trends Survey 5 Cycles 1 to 4 (2017-2020)

This supplementary material has been provided by the authors to give readers additional information about their work.

© 2023 Stephens ES et al. *JAMA Network Open*.

eTable 1. Survey Questions Used to Ascertain Study Measures, Health Information Trends Survey 5 Cycles 1 to 4 (2017-2020)

Measure	Questionnaire	Response options
**HPV Awareness**		
HPV awareness	Have you ever heard of HPV? HPV stands for human papillomavirus. It is not HIV, HSV, or herpes.	• Yes<br>• No
HPV vaccine awareness	A vaccine to prevent HPV infection is available and is called the HPV shot, cervical cancer vaccine, GARDASIL®, or Cervarix®. Before today, have you ever heard of the cervical cancer vaccine or HPV shot?	• Yes<br>• No
**HPV Knowledge**		
Knowledge that HPV can cause cervical cancer	Do you think HPV can cause cervical cancer? (among participants who responded “yes” to having heard of HPV)	• Yes<br>• No<br>• Not Sure<br>• Skip
Knowledge that HPV can cause penile cancer	Do you think HPV can cause penile cancer? (among participants who responded “yes” to having heard of HPV)	• Yes<br>• No<br>• Not Sure<br>• Skip
Knowledge that HPV can cause anal cancer	Do you think HPV can cause anal cancer? (among participants who responded “yes” to having heard of HPV)	• Yes<br>• No<br>• Not Sure<br>• Skip
Knowledge that HPV can cause oropharyngeal cancer	Do you think HPV can cause oral cancer? (among participants who responded “yes” to having heard of HPV)	• Yes<br>• No<br>• Not Sure<br>• Skip
**Educational Attainment**		
Educational attainment	What is the highest level of schooling you completed?	• Less than 8 years<br>• 8 through 11 years<br>• 12 years or completed high school<br>• Post high school training other than college (vocational or technical)<br>• Some college<br>• College graduate<br>• Postgraduate<br>• Skip
**Race and Ethnicity**		
Race	What is your race?	• White<br>• Black or African American<br>• American Indian or Alaska Native<br>• Asian Indian<br>• Chinese<br>• Filipino<br>• Japanese<br>• Korean<br>• Vietnamese<br>• Other Asian<br>• Native Hawaiian<br>• Guamanian or Chamorro<br>• Samoan<br>• Other Pacific Islander
Ethnicity	Are you of Hispanic, Latino/a, or Spanish origin?	• Yes<br>• No<br>• Skip

HPV= human papillomavirus, HIV=human immunodeficiency virus, HSV=herpes simplex virus.

eTable 2. Comparison of Participants With and Without Data on Race and Ethnicity

	**Among Participants with Data on Race and Ethnicity (N=14,444)**		**Among All Participants Regardless of Missing Data on Race and Ethnicity (N= 16,092)**	
**Characteristic**	**n**	**Weighted % (95% CI)**	**n**	**Weighted % (95% CI)**
Age Group, Years				
18-34	1,882	24.5 [23.2,25.8]	1,944	23.4 [22.2,24.6])
35-44	1,840	16.4 [15.3,17.5]	1,906	15.6 [14.6,16.6])
45-54	2,343	23.5 [22.4,24.6]	2,484	22.9 [21.9,23.9])
55-64	3,288	16.2 [16.0,16.4]	3,580	16.2 [16.1,16.3])
65-75	3,300	11.7 [11.5,12.0]	3,704	12.1 [11.9,12.3])
76-104	1,572	6.3 [6.0,6.5]	1,967	7.2 [7.1,7.4])
. (missing)	219	1.5 [1.2,1.8]	507	2.7 [2.3,3.1]
Median (IQR)		57(43,68)		52 (37, 63)
Sex				
Female	8,361	50.6 [50.2,51.0]	9,245	50.3 [50.0,50.5]
Male	5,934	48.5 [48.2,48.9]	6,494	47.9 [47.6,48.1]
. (missing)	149	0.9 [0.7,1.1]	353	1.9 [1.6,2.2]
Education				
College graduate or more	6,666	31.9 [31.7,32.2]	6,987	30.3 [30.2,30.5]
Some college	4,274	38.3 [37.5,39.1]	4,653	37.2 [36.4,38.0]
High school graduate	2,502	21.6 [20.7,22.4]	2,898	22.3 [21.5,23.1]
Less than high school	913	7.7 [6.9,8.5]	1,099	8.0 [7.2,8.8]
. (missing)	89	0.5 [0.3,0.9]	455	2.2 [1.9,2.6]
Annual Household Income				
$75,000+	5,575	40.3 [38.9,41.7]	5,813	38.2 [36.9,39.5]
$35,000 to $74,999	4,447	31.4 [30.0,32.9]	4,857	31.1 [29.7,32.5]
$0 to $34,999	4,362	27.8 [26.5,29.0]	5,182	29.2 [28.0,30.5]
. (missing)	120	0.5 [0.3,0.9]	240	1.5 [1.1,1.9]
Marital Status				
Divorced/ widowed/ separated	4,046	14.0 [13.5,14.5]	8,323	14.6 [14.2,15.1]
Married/living as married	7,827	54.8 [54.2,55.4]	2,644	53.2 [52.7,53.7]
Single	2,451	30.4 [30.0,30.8]	4,636	29.7 [29.4,29.9]
. (missing)	120	0.8 [0.6,1.0]	489	2.5 [2.1,2.9]
Sexual Orientation				
Gay/ lesbian/ bisexual	574	4.7 [4.0,5.5]	597	4.5 [3.9,5.3]
Heterosexual	13,364	91.9 [91.0,92.7]	14,272	89.0 [88.1,89.8]
Other	179	1.5 [1.1,1.9]	219	1.5 [1.2,2.0]
. (missing)	327	2.0 [1.7,2.3]	1,004	5.0 [4.5,5.5]
Insurance Status				
No	735	8.4 [8.1,8.7]	832	8.4 [8.4,8.5]
Yes	13,539	90.4 [90.0,90.8]	15,003	90.1 [89.8,90.4]
. (missing)	170	1.2 [0.9,1.5]	257	1.5 [1.2,1.8]

CI = confidence interval; IQR = interquartile range; NH = Non-Hispanic.

© 2023 Stephens ES et al. *JAMA Network Open*.

eTable 3. Sociodemographic Characteristics by Race and Ethnicity, Health Information Trends Survey 5 Cycles 1 to 4 (2017-2020)

	**Overall (N= 14,444)**		**Hispanic (N=2,214)**		**Asian (N= 661)**		**Black (N=2,011)**		**Other (N=520)**		**White (N=9,038)**	
**Characteristic**	**n**	**Weighted % (95% CI)**	**n**	**Weighted % (95% CI)**	**n**	**Weighted % (95% CI)**	**n**	**Weighted % (95% CI)**	**N**	**Weighted % (95% CI)**	**n**	**Weighted % (95% CI)**
Age Group, Years (n=14,225)												
18-34	1,882	24.9 [23.6,26.2]	404	32.2 [28.9,35.8]	130	34.4 [28.1,41.3]	199	19.7 [16.5,23.4]	102	41.3 [32.9,50.3]	1,047	22.3 [20.8,23.9]
35-44	1,840	16.6 [15.6,17.8]	378	21.0 [18.1,24.2]	124	21.1 [16.8,26.3]	250	20.1 [17.0,23.6]	77	13.1 [9.4,17.8]	1,011	14.7 [13.5,16.1]
45-54	2,343	23.8 [22.7,24.9]	413	26.5 [23.3,29.8]	117	23.6 [18.5,29.6]	386	29.0 [25.5,32.8]	112	25.4 [19.7,32.2]	1,315	22.2 [20.9,23.5]
55-64	3,288	16.4 [16.2,16.7]	446	10.8 [9.5,12.2]	110	9.8 [7.6,12.6]	532	17.2 [15.4,19.1]	109	12.9 [9.4,17.4]	2,091	18.4 [18.1,18.8]
65-75	3,300	11.9 [11.6,12.1]	369	6.4 [5.6,7.4]	114	6.6 [5.1,8.6]	430	10.0 [8.7,11.4]	88	5.7 [3.9,8.1]	2,299	14.3 [13.9,14.7]
76-104	1,572	6.4 [6.1,6.6]	162	3.1 [2.6,3.8]	58	4.3 [3.0,6.2]	165	4.0 [3.2,5.0]	24	1.6 [0.8,3.2]	1,163	8.0 [7.6,8.4]
Median (IQR)		57(43,68)		52(38,64)		51(37,65)		57(45,66)		52(37,63)		60(45,70)
Sex (n= 14,295)												
Female	8,361	51.0 [50.7,51.4]	1,272	50.7 [47.8,53.5]	333	48.1 [42.3,53.9]	1,339	57.3 [53.3,61.2]	318	42.6 [35.1,50.5]	5,099	50.8 [50.3,51.2]
Male	5,934	49.0 [48.6,49.3]	915	49.3 [46.5,52.2]	326	51.9 [46.1,57.7]	640	42.7 [38.8,46.7]	198	57.4 [49.5,64.9]	3,855	49.2 [48.8,49.7]
Education (n=14,355)												
College graduate or more	6,666	32.1 [31.8,32.4]	684	21.2 [19.4,23.0]	455	58.3 [52.4,64.0]	743	26.1 [23.7,28.6]	228	25.9 [20.1,32.7]	4,556	34.0 [33.7,34.4]
Some college	4,274	38.5 [37.7,39.3]	681	36.8 [33.8,39.9]	118	23.9 [18.0,31.0]	662	34.4 [31.2,37.8]	171	39.6 [30.8,49.0]	2,642	40.8 [39.9,41.7]
High school graduate	2,502	21.7 [20.8,22.5]	459	25.2 [22.3,28.2]	52	11.8 [8.3,16.6]	426	27.9 [24.7,31.4]	82	28.4 [19.7,39.2]	1,483	20.2 [19.2,21.3]
Less than high school	913	7.7 [6.9,8.5]	365	16.9 [14.4,19.8]	32	6.0 [3.6,10.0]	165	11.6 [8.8,15.3]	38	6.1 [3.8,9.9]	313	4.9 [4.1,5.8]
Annual Household Income (n=14,384)												
$75,000+	5,575	40.5 [39.1,41.9]	629	31.2 [27.7,34.9]	325	50.1 [44.2,56.1]	457	23.9 [20.7,27.5]	167	38.7 [30.1,47.9]	3,997	44.9 [43.3,46.6]
$35,000-$74,999+	4,447	31.6 [30.1,33.1]	684	35.0 [31.6,38.6]	188	28.6 [23.0,34.8]	592	30.0 [26.4,33.9]	180	37.1 [29.8,45.1]	3,997	31.0 [29.3,32.8]
$0 to $34,999	4,362	27.9 [26.7,29.2]	880	33.8 [30.5,37.4]	144	21.3 [16.1,27.5]	949	46.1 [41.9,50.3]	166	24.2 [17.8,32.1]	2,223	24.1 [22.6,25.6]
Marital Status (n=14,324)												
Divorced/widowed/ separated	4,046	14.1 [13.6,14.6]	574	11.3 [9.9,13.0]	88	5.6 [4.0,7.9]	695	15.8 [13.2,18.7]	148	11.0 [8.0,14.9]	2,541	15.4 [14.9,16.0]

© 2023 Stephens ES et al. *JAMA Network Open*.

	Overall (N= 14,444)		Hispanic (N=2,214)		Asian (N= 661)		Black (N=2,011)		Other (N=520)		White (N=9,038)	
**Characteristic**	**n**	**Weighted % (95% CI)**	**n**	**Weighted % (95% CI)**	**n**	**Weighted % (95% CI)**	**n**	**Weighted % (95% CI)**	**N**	**Weighted % (95% CI)**	**n**	**Weighted % (95% CI)**
Married/living as married	7,827	55.2 [54.6,55.8]	1,182	52.7 [49.5,55.9]	451	62.5 [55.4,69.1]	692	35.0 [31.6,38.6]	244	45.7 [37.6,54.2]	5,258	59.2 [58.5,59.8]
Single	2,451	30.6 [30.2,31.0]	419	36.0 [32.9,39.3]	120	31.9 [25.3,39.3]	604	49.2 [45.7,52.7]	126	43.3 [34.6,52.4]	1,182	25.4 [24.9,25.9]
Sexual Orientation (n=14,117)												
Gay/ lesbian/ bisexual	574	4.8 [4.1,5.6]	88	6.3 [4.4,9.0]	26	7.6 [4.4,12.9]	58	3.9 [2.7,5.6]	38	6.5 [3.6,11.4]	364	4.3 [3.5,5.2]
Heterosexual	13,364	93.7 [92.9,94.5]	1,992	91.9 [89.1,94.0]	584	90.0 [84.8,93.6]	1,844	93.8 [91.9,95.3]	457	87.5 [78.7,93.1]	8,487	94.7 [93.7,95.6]
Other	179	1.5 [1.1,2.0]	41	1.8 [1.1,3.1]	18	2.4 [1.2,4.7]	40	2.2 [1.5,3.4]	14	5.9 [2.0,16.2]	66	1.0 [0.6,1.6]
Insurance Status (n= 14,274)												
No	735	8.5 [8.2,8.8]	236	16.0 [13.4,19.1]	19	5.0 [1.7,13.3]	119	10.4 [7.5,14.1]	36	8.4 [5.0,13.8]	325	6.6 [5.8,7.4]
Yes	13,539	91.5 [91.2,91.8]	1,943	84.0 [80.9,86.6]	634	95.0 [86.7,98.3]	1,867	89.6 [85.9,92.5]	476	91.6 [86.2,95.0]	8,619	93.4 [92.6,94.2]

CI = confidence interval; IQR = interquartile range; “n” indicates the number of individuals for each level of characteristic. “N” indicates the total number of individuals in each population of interest (e.g., all Hispanic individuals, all non-Hispanic Asian individuals, etc.). Other racial and ethnic group includes American Indian or Alaska Native, Native Hawaiian or other Pacific Islander, and multiple race.

© 2023 Stephens ES et al. *JAMA Network Open*.

eFigure 1. Data Inclusion by Educational Level and Race and Ethnicity, Health Information Trends Survey 5 Cycles 1 to 4 (2017-2020)

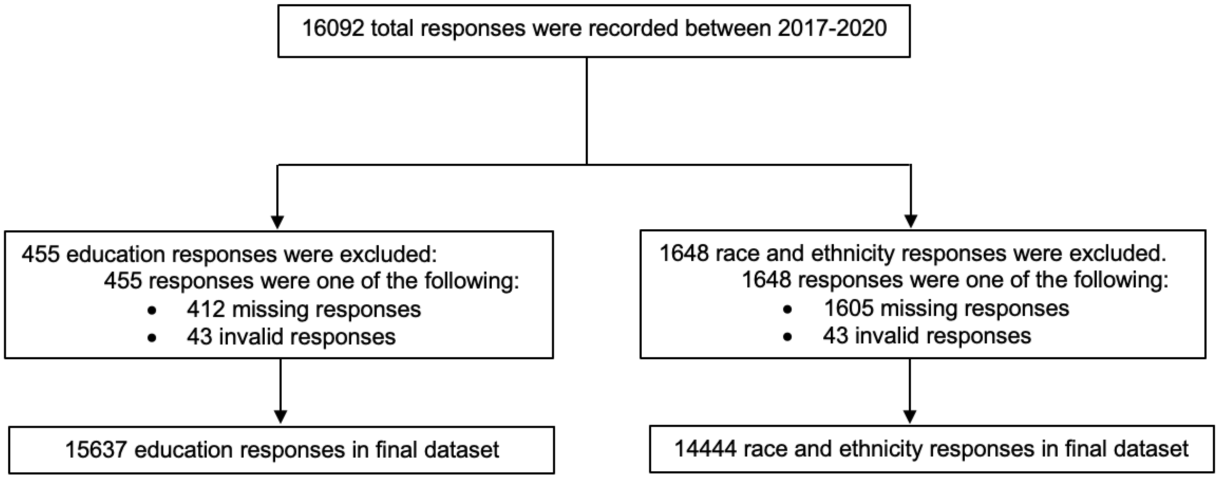

© 2023 Stephens ES et al. *JAMA Network Open*.

eFigure 2. Trends in Knowledge that HPV Can Cause Penile, Anal, and Oropharyngeal Cancer Among Participants Who Were Aware of HPV, Health Information Trends Survey 5 Cycles 1 to 4 (2017-2020)

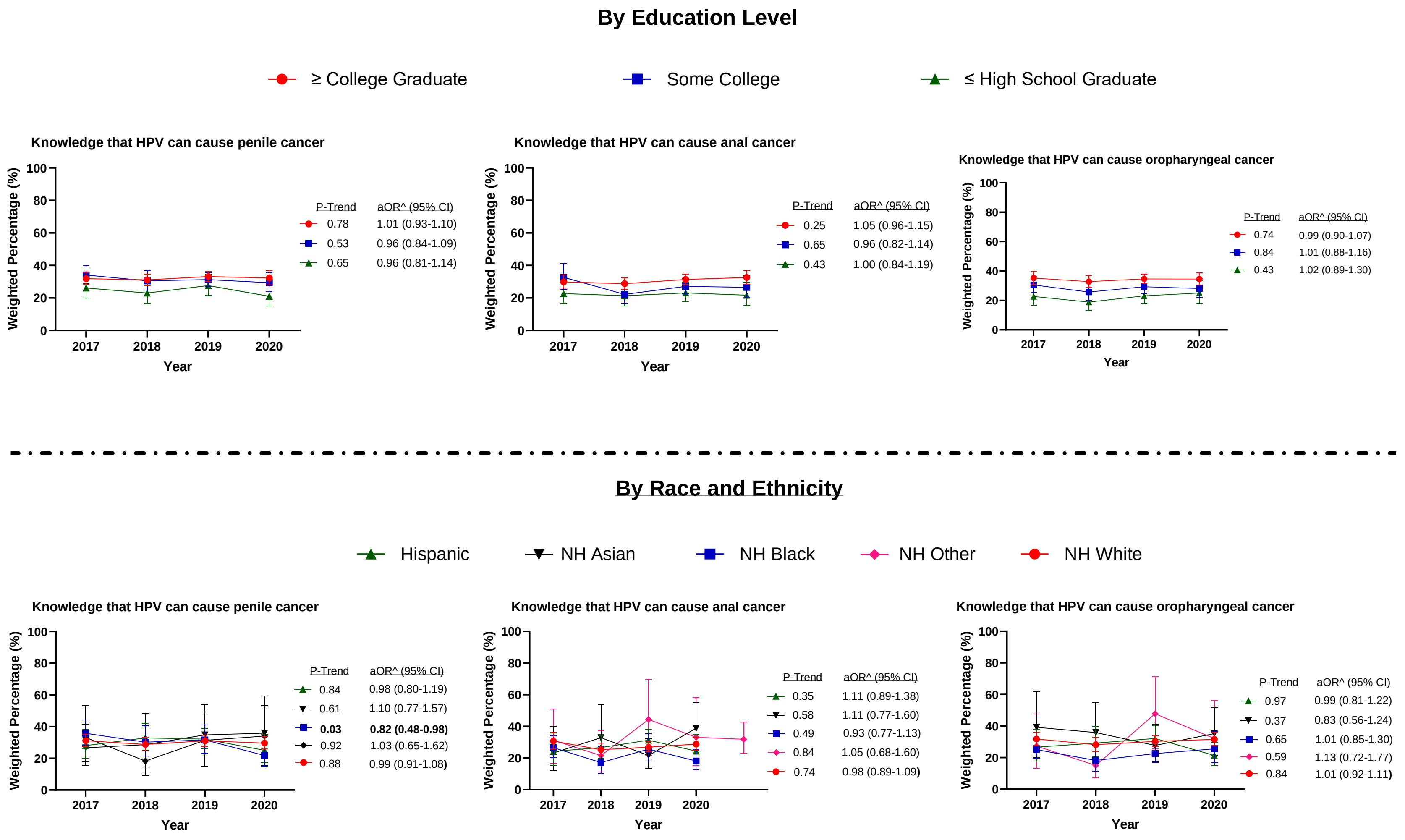

Adjusted odds ratios indicate the relative change in prevalence per year. aOR = adjusted odds ratio; CI = confidence interval; HPV = human papillomavirus; NH = non-Hispanic. NH-Other racial and ethnic group includes American Indian or Alaska Native, Native Hawaiian or other Pacific Islander, and multiple race.

© 2023 Stephens ES et al. *JAMA Network Open*.
