# Supplementary material for: Human Papillomavirus Awareness by Educational Level and by Race and Ethnicity
Source: JAMA Netw Open. 2023 Nov 14;6(11):e2343325. doi: 10.1001/jamanetworkopen.2023.43325 (PMC10646733; doi:10.1001/jamanetworkopen.2023.43325)
Supplement: Supplement 2. — Data Sharing Statement [file jamanetwopen-e2343325-s002.pdf]

Data Sharing Statement

Stephens. Human Papillomavirus Awareness by Educational Level and by Race and Ethnicity. *JAMA Netw Open*. Published November 14, 2023. doi:10.1001/jamanetworkopen.2023.43325

Data
Data available: No

Additional Information
Explanation for why data not available: The data are publicly available on the Health Information National Trends Survey website: https://hints.cancer.gov/data/Default.aspx
